# Genomic Characterization of Colistin-Resistant Isolates from the King Fahad Medical City, Kingdom of Saudi Arabia

**DOI:** 10.3390/antibiotics11111597

**Published:** 2022-11-11

**Authors:** Liliane Okdah, Mohammed Saeed AlDosary, Abeer AlMazyed, Hussain Mushabbab Alkhurayb, Meshari Almossallam, Yousef sultan Al Obaisi, Mohammed Ali Marie, Tamir Abdelrahman, Seydina M. Diene

**Affiliations:** 1 King Abdullah International Medical Research Center, National Guard Health Affairs, Riyadh 1126, Saudi Arabia; 2 Pathology and Clinical Laboratory Medicine Administration, King Fahad Medical City, Riyadh 11525, Saudi Arabia; 3 Clinical Laboratory Sciences Department, College of Applied Medical Sciences, King Saud University, Riyadh 11433, Saudi Arabia; 4 Laboratoire National de Santé (LNS), F3XP+Q4 Dudelange, Luxembourg; 5 MEPHI, IRD, APHM, IHU-Mediterranee Infection, Faculté de Pharmacie, Aix-Marseille University, 13005 Marseille, France

**Keywords:** *Acinetobacter baumannii*, *Klebsiella pneumoniae*, *Pseudomonas aeruginosa*, colistin resistance, ST2 clone, circulating sub-clones, comparative genomics

## Abstract

Background: Whole-genome sequencing is one of the best ways to investigate resistance mechanisms of clinical isolates as well as to detect and identify circulating multi-drug-resistant (MDR) clones or sub-clones in a given hospital setting. Methods: Here, we sequenced 37 isolates of *Acinetobacter baumannii*, 10 *Klebsiella pneumoniae*, and 5 *Pseudomonas aeruginosa* collected from the biobank of the hospital setting of the King Fahad Medical City. Complete phenotypic analyses were performed, including MALDI-TOF identification and antibiotic susceptibility testing. After the genome assembly of raw data, exhaustive genomic analysis was conducted including full resistome determination, genomic SNP (gSNP) analysis, and comparative genomics. Results: Almost all isolates were highly resistant to all tested antibiotics, including carbapenems and colistin. Resistome analysis revealed many antibiotic resistance genes, including those with resistance to β-lactams, aminoglycosides, macrolides, tetracyclines, sulfamids, quinolones, and phenicols. In *A. baumannii* isolates, the endemic carbapenemase *bla*_OXA-23_ gene was detected in 36 of the 37 isolates. Non-synonymous mutations in *pmrB* were detected in almost all of the isolates and likely mediated colistin resistance. Interestingly, while classical analyses, such as MLST, revealed the predominance of an ST2 clone in *A. baumannii* isolates, the genomic analysis revealed the presence of five circulating sub-clones and identified several isolate transmissions between patients. In the 10 *K. pneumoniae* isolates, several resistance genes were identified, and the observed carbapenem resistance was likely mediated by overexpression of the detected extended-spectrum-β-lactamase (ESBL) genes associated with low membrane permeability as few carbapenemase genes were detected with just *bla*_OXA-48_ in three isolates. Colistin resistance was mediated either by non-synonymous mutations in the MgrB regulator, PmrA, PmrB, and PhoQ proteins or the presence of the MCR-1 protein. Here, gSNP analysis also revealed the existence of bacterial clones and cases of isolate transmissions between patients. The five analyzed *P. aeruginosa* isolates were highly resistant to all tested antibiotics, including carbapenems mediated by loss or truncated OprD porin, and colistin resistance was associated with mutations in the genes encoding the PmrA, PmrB, or PhoQ proteins. Conclusion: We demonstrate here the usefulness of whole-genome sequencing to exhaustively investigate the dissemination of MDR isolates at the sub-clone level. Thus, we suggest implementing such an approach to monitor the emergence and spread of new clones or sub-clones, which classical molecular analyses cannot detect. Moreover, we recommend increasing the surveillance of the endemic and problematic colistin resistance *mcr*-1 gene to avoid extensive dissemination.

## 1. Introduction

The emergence and dissemination of antibiotic resistance, especially in Gram-negative bacteria (GNB), is a major global public health issue [1]. Nosocomial infections caused by multi-drug-resistant (MDR) bacteria have led to increased mortality, morbidity, and cost of treatment and continue to endanger the lives of patients, especially the immunocompromised in hospitals [1]. Carbapenem drugs, including imipenem, meropenem, doripenem, and ertapenem, are used as last-resort antibiotics for the treatment of hospital-acquired infections. Prescriptions of these drugs have increased, leading to the emergence and spread of MDR isolates producing VIM—Verona imipenemase, IMP—imipenemase, OXA—oxacillinase, KPC—*Klebsiella pneumoniae* carbapenemase, and NDM—New Delhi metallo-β-lactamase [2,3]. This worldwide issue of MDR mediated by these β-lactamase enzymes has led to the reuse of drugs such as polymyxins because of their nephrotoxicity and neurotoxicity in humans [4]. Polymyxins such as colistin are cationic antimicrobial peptides synthetized by *Bacillus polymyxa*, which was discovered in 1947 [5]. The latter is nowadays administrated in aerosol to treat nosocomial pneumopathies in patients with cystic fibrosis and also in the context of digestive decontamination protocols due to the lack of intestinal absorption [6]. Colistin is a bactericidal polypeptide polycationic antibiotic that targets and interacts with the negatively charged lipopolysaccharide (LPS), therefore disrupting the bacterial membrane [7]. However, the introduction of colistin in clinical practices has resulted in the emergence and spread of colistin resistance in clinical settings worldwide [8]. The well-known and initially described resistance mechanisms to colistin were mediated by mutations in chromosomic genes, leading to the activation of two-component systems (i.e., PhoP/PhoQ and PmrA/PmrB) which results in sugar biosynthesis, such as phosphoethanolamine (PEtN) or 4 amino-4-deoxy-L-arabinose (L-Ara4N) and their addition to the lipid A moiety of LPSs [9]. In November 2015, Liu et al. reported the first detection of a plasmid-mediated colistin resistance *mcr-1* gene [10]. This discovery was followed by screening for *mcr* genes in samples from all over the world and from various human and animal origins and by the description of different variants of these genes [7,11,12].

Consequently, reports on colistin resistance are increasing over the world in clinical settings, animals, food, and the environment [7,13]. In Riyadh, before this study, only extended-spectrum beta-lactamase (ESBL)-producing *Enterobacteriaceae,* including *Escherichia coli* and *Klebsiella pneumoniae,* have been reported in this region [14]. The approach of combining high-throughput sequencing and bioinformatics has demonstrated its usefulness in investigating any antibiotic resistance concern [15,16]. Thus, here, we conducted a genomic characterization of clinical isolates collected at the King Fahad Medical City (KFMC) by whole-genome sequencing (WGS) to figure out their resistance mechanisms to antibiotics and investigate circulating clones within this hospital setting.

## 2. Results

### 2.1. Clinical Bacterial Isolates

A total of 52 isolates dating back to 2012 were collected and stored based on their colistin resistance phenotype and consisted of three bacterial species, including *Acinetobacter baumannii* (*n* = 37 isolates), *Klebsiella pneumoniae* (*n* = 10 isolates), and *Pseudomonas aeruginosa* (*n* = 5 isolates). As presented in Supplementary Tables S1 and S2, all isolates were collected from different rooms or care units of the KFMC and twelve different clinical samples, with the majority (30 out of 52 isolates) being from pulmonary samples (i.e., sputum and endotracheal), and the other samples included cerebrospinal fluid, the central venous line, blood, the peripheral line left, bedsores, wounds, tissues, indwelling catheters, urine, and rectal swabs. In total, 56% of the isolates (*n* = 27) were collected from male patients and the 44% of the isolates (*n* = 25) were from female patients. All patients were adults and aged from 18 years to 89 years old, except one patient who was 13 years old. After bacterial culture on a MacConkey agar plate, all isolates were identified by MALDI-TOF with a score identification of more than 2.30, confirming their identification at the species level.

### 2.2. Antibiotic Susceptibility Testing (AST)

As presented in Supplementary Table S1, the AST reveals that all *A. baumannii* and *P. aeruginosa* isolates were almost resistant to all tested antibiotics. Indeed, we observed 100% resistance to all tested β-lactams, including ceftazidime, cefepime, ticarcillin/tazobactam, meropenem, and imipenem for all isolates of both species. Additionally, 100% resistance to trimethoprim/sulfamethoxazole and colistin was observed for both species. Moreover, the performed AST by microdilution reveals that colistin MICs for *A. baumannii* isolates ranged from 8 µg/mL to 128 µg/mL, whereas those of *P. aeruginosa* ranged from 4 µg/mL to 128 µg/mL (Supplementary Table S1). Only susceptibility to aminoglycosides such as gentamycin and amikacin was observed. For *K. pneumoniae* isolates, full resistance was observed against the different families of antibiotics tested, including β-lactams, aminoglycosides, trimethoprim/sulfamethoxazole, quinolones, tigecycline, levofloxacin, and colistin. Susceptibility was observed for only cefepime and gentamicin in two *K. pneumoniae* isolates. The microdilution assay for colistin revealed MICs ranged between 8 µg/mL and 128 µg/mL (Supplementary Table S2).

### 2.3. Genome Assembly Results

The genome assembly with the A5-miseq pipeline resulted in an average number of 203 contigs for all isolates; the smallest number of contigs was obtained for a *K. pneumoniae* isolate, KP02 (*n* = 72 contigs), and the higher number of contigs was obtained for *P. aeruginosa* PA191 (*n* = 1484 contigs) (Supplementary Table S3). Based on the obtained depth of coverage, all the genomes were sufficiently sequenced with at least 14X coverage for the PA193 isolate and 175X max coverage for the AB54 isolate, and the average depth of coverage was 80X. The average genome size of *A. baumannii* isolates was estimated to be 3’933’843-bp, 5’742’318-bp for *K. pneumoniae* isolates, and 6’671’219-bp for *P. aeruginosa* isolates. The average %GC was 38.92%, 56.79%, and 66% for *A. baumannii*, *K. pneumoniae*, and *P. aeruginosa* isolates, respectively. All WGS data have been deposited in the GenBank database, and the accession number of each genome is given in Supplementary Table S3. 

### 2.4. Investigation of Genes and Gene Mutations Associated with the Antibiotic Resistance Phenotype (The Resistome) from the WGS Data

***A. baumannii***: According to the observed high-resistance phenotype, the full resistome of all 37 *A. baumannii* isolates was identified. Table 1 presents 11 types of β-lactamase enzymes, including ADC-73, ADC-30, TEM-1D, BlaA1, BlaA2, Mbl, OXA-23, OXA-66, OXA-72, OXA-181, and Zn_hydrolase, which have been identified in these isolates. Interestingly, the endemic carbapenemase *bla*_OXA-23_ gene was detected in almost all isolates except one that harbored the carbapenemase *bla*_OXA-66_ variant (Table 1). Nine types of aminoglycoside resistance genes were also identified, and the most prevalent genes were the aminoglycoside resistance methylase *armA*, the aminoglycoside 3’-phosphotransferases (*aph(3’)-Ia* and *aphA*6), and the streptomycin resistance genes (*strA* and *strB*) (Table 1). Almost all isolates harbored resistance genes to macrolides (*mphE* and *msrE*), tetracyclines (*tetB*), and sulfonamides (*sulI*). Thanks to the high level of colistin resistance observed in these *A. baumannii* isolates, chromosomal gene mutations in colistin resistance genes, including *pmrA*, *pmrB*, *lpxA*, *lpxC*, and *lpxD*, were analyzed. As expected, while no mutation was observed in *pmrA* and *lpxACD* genes, non-synonymous mutations in the *pmrB* gene were observed in all isolates, and interestingly, one amino acid (AA) substitution (A138T) in PmrB was observed in 36 out of the 37 isolates (Table 1) suggestive of clonal dissemination within the hospital. 

***K. pneumoniae***: As observed for *A. baumannii* isolates, many antibiotic-resistance genes were detected in *K. pneumoniae* isolates (Table 2). Based on the observed phenotype, resistance to penicillin and cephalosporin classes was mediated by the presence of ESBLs such as SHV and CTX-M types (Table 2). Surprisingly, while resistance phenotypes to carbapenems (ertapenem, meropenem, and imipenem) were observed, only *bla*_OXA-48_ carbapenemase was detected in three *K. pneumoniae* isolates, suggesting that the observed carbapenem resistance was mediated by other mechanisms, such as low permeability associated with overexpression of ESBL enzymes. Resistance to aminoglycosides was mediated by the presence of different enzymes, including mainly AadA2, Ant3’Th, ArmA, and Sat-2A (Table 2). As shown in Table 2, diverse genes conferring resistance on quinolones, macrolides, phenicols, fosfomycin, sulfonamides, tetracyclines, and trimethoprim were identified. Regarding colistin resistance, contrary to *A. baumannii* isolates, resistance to this antibiotic was mediated by diverse mechanisms, including MgrB amino acid substitution (Q22P) or inactivation of MgrB in four isolates, and amino acid substitution in PmrA (G53V), in PmrB (T128P, T157P), or in PhoQ (L52V, G385S, and D495-) (Table 2). 

**Figure 1:**
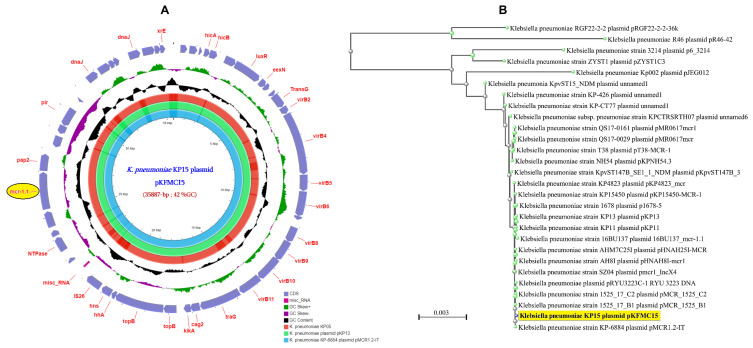
The *K. pneumoniae* KP15 plasmid pKFMC15 harboring the *mcr*-1.1. (**A**) The circular representation of this plasmid and its comparison with the closest plasmid sequences retrieved from the NCBI database. As shown in this figure, the same plasmid was identified in the *K. pneumoniae* KP05 strain isolated from the same hospital. (**B**) The phylogenetic relationship with the most closely related plasmids from the NBCI database.

Worryingly, the mobilized colistin resistance gene *mcr*-1 was detected in two *K. pneumoniae* isolates, suggesting a putative beginning of the spread of this gene within this hospital setting. Indeed, as shown in Figure 1, our analysis revealed that this gene was located on a recombinant plasmid of 35.89-Kb and 42%GC, which is detected in both *mcr*-1-positive isolates (KP15 and KP5 isolates) (Figure 1A). Moreover, the BlastN phylogenetic tree analyses against plasmid sequences from the NCBI database revealed high similarity with other *mcr*-1-harboring plasmids reported elsewhere, such as the *K. pneumoniae* plasmid pMCR1.2-IT reported in Italy [17] (Figure 1B). 

***P. aeruginosa***: As seen for the two other species of this study, the resistance phenotype exhibited by *P. aeruginosa* isolates was mediated by various resistance genes, including five β-lactamase genes (*bla*_AQU1,_ *bla*_OXA-10_, *bla*_OXA-50_, *bla*_GES-1_, and *bla*_VEB-1_) conferring resistance on penicillins and cephalosporins (Table 3). The resistance observed in the five *P. aeruginosa* isolates against carbapenems (meropenem and imipenem) was induced by loss or deleterious mutations of the gene that encodes the OprD porin, which is associated with a high level of carbapenem resistance in this bacterial species. Aminoglycoside’s resistance was related to the presence of five different genes, including *aadA*1, *aadB*, *aphA*15, *aph*3-Ib, and *aph*6-I (Table 3). Resistance to quinolones was mediated by the presence of four copies of the *oqxBgb* and *qepA* genes in the five isolates, while the *catB*7 gene was responsible for the phenicol resistance, and *sulI*, *tetA*, *tcr*-3, and *tetR* were responsible for the resistance to sulfonamides and tetracyclines, respectively (Table 3). Interestingly, sequence analysis revealed that the colistin resistance in these *P. aeruginosa* isolates was associated with non-synonymous mutations in the chromosomal *pmrA, pmrB*, and *phoQ* genes (Table 3). As shown in Table 3, the most amino acid substitutions were observed in the PmrB sensor protein, including S2P, A4T, V15I, G68S, which were identified in most of the *P. aeruginosa* isolates. The PhoQ sensor was truncated in one of the isolates (i.e., the PA193 isolate).

### 2.5. Genotype, Comparative Genomics, and Phylogenomic Relationship of Isolates

**Figure 2:**
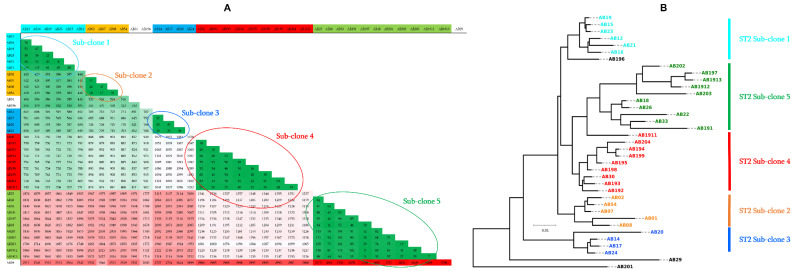
Genomic characterization of clinical *A. baumannii* isolates. (**A**) Pairwise comparison of genomic gSNPs of *A. baumannii* isolates demonstrating the existence of five circulating sub-clones in this hospital setting. (**B**) Whole-proteome-based phylogenetic tree of *A. baumannii* isolates highlighting the observed sub-clones.

***A. baumannii***: The MLST analysis based on the seven housekeeping genes (*cpn*60, *fusA*, *gltA*, *pyrG*, *recA*, *rplB*, and *rpoB*) revealed that 35 out of the 37 isolates (98%) belonged to the ST2 type, 1 isolate was ST604 and 1 was ST45 (Table 1). Moreover, using the WGS data, further investigations of the clonality of isolates by pairwise comparison of the number of core genome SNPs (gSNPs) interestingly revealed five sub-clones among the isolates. Indeed, as shown in Figure 2A, these five groups of isolates can be observed. The sub-clones 1, 2, and 3 were composed of six, four, and four isolates sharing less than 117, 37, and 50 gSNPs, respectively, whereas the two remaining sub-clones 4 and 5 included more isolates and were composed of nine and eleven isolates sharing less than 63 and 116 gSNPs, respectively (Figure 2A). Three isolates (i.e., AB1, AB196, and AB29) were not associated with any sub-clone. Interestingly, in group 1 (sub-clone 1), three isolates (AB15, AB19, and AB23) shared less than 21 gSNPs, suggesting that the same isolate was transmitted between the patients. In group 2 (sub-clone 2), the three isolates (AB02, AB07, and AB54) can be considered as the same isolate thanks to the minimal number of gSNPs shared between them (≤18 gSNPs) (Figure 2A). In group 4 (sub-clone 4), the two pairs of isolates AB30/AB192/AB193 and AB194/AB195/AB199 appeared as two single isolates also transmitted between patients thanks to the small number of gSNPs shared. In group 5 (sub-clone 5), the pair of isolates AB26/AB18 and AB197/AB1912/AB1913 also appeared to be a case of transmission between patients. To confirm the existence of the identified sub-clones, we performed a phylogenetic tree analysis based on the whole proteomes of all *A. baumannii* isolates. As expected, the inferred full proteome-based phylogenetic tree globally confirmed the five sub-clones detected in the first analysis (Figure 2B), and only some isolates appeared to be not correctly clustered.

**Figure 3:**
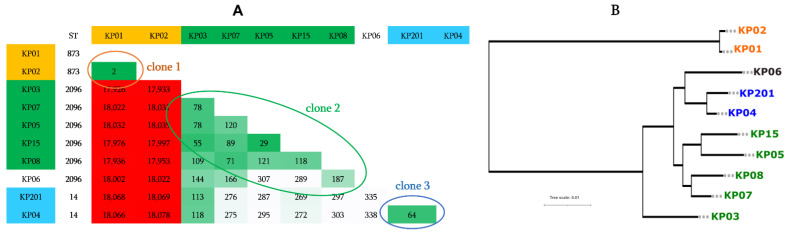
Genomic characterization of *K. pneumoniae* isolates. (**A**) Pairwise comparison of genomic gSNPs of *K. pneumoniae* showing the three distinguished sub-clones; (**B**) whole-proteome-based phylogenetic tree of *K. pneumoniae* isolates.

***K. pneumoniae***: MLST analysis of these *K. pneumoniae* isolates revealed three different ST types, including ST873 (*n* = two isolates), ST2096 (*n* = six isolates), and ST14 (*n* = two isolates) (Figure 3A). The performed pairwise gSNPs comparison was in concordance with the MLST analysis results, which confirm the existence of three bacterial clones and one case of isolate transmission between patients (i.e., KP01 and KP02) since these two isolates had only two gSNPs (Figure 3A). As expected, the performed whole-proteome-based phylogenetic tree confirmed the existence of the three clones among these *K. pneumoniae* isolates (Figure 3B).

**Figure 4:**
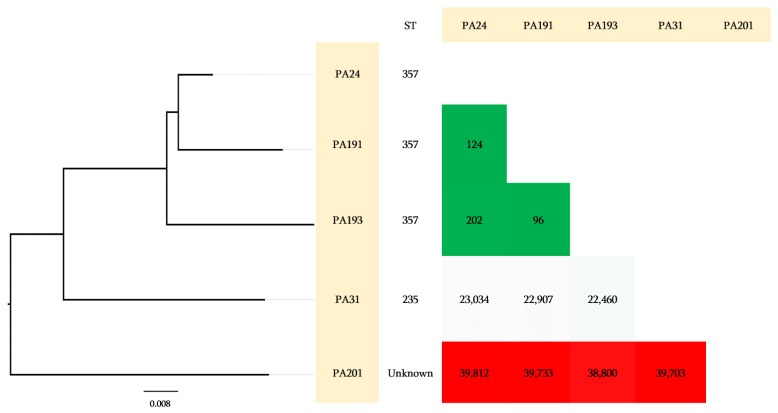
Genomic characterization of *P. aeruginosa* isolates and the pairwise comparison of the genomic gSNPs.

***P. aeruginosa***: For *P. aeruginosa* isolates, shown in Figure 4, three out of the five isolates were identified as ST357, one isolate as ST235, and the one remaining isolate was an unknown ST. The number of gSNPs was significantly smaller between ST357 isolates compared to the two remaining isolates (Figure 4). 

## 3. Discussion

Nosocomial infections associated with MDR bacteria such as GNB, specifically *Enterobacteriaceae, Pseudomonas*, and *Acinetobacter*, are a major world health problem that have not only led to an increase in mortality, morbidity, and cost of treatment, but also continue to endanger the lives of ill patients, especially immunocompromised patients in hospital settings [1]. Nowadays, colistin represents one of the few viable antimicrobial drugs that may be used against aggressive infections due to MDR bacteria, and the emergence of plasmid-mediated colistin resistance since 2015 in Gram-negative bacteria has seriously compromised its use [18]. Indeed, as described in the literature, the prevalence of colistin resistance is increasingly reported in several countries worldwide. This resistance is associated either with chromosomal gene mutations or the transmission of plasmid-mediated colistin resistance *mcr*-1 [19,20]. At the local level, the interest in colistin resistance tracking was highlighted in a 2017 review by Kharaba et al., who shed light on the alarming emergence in colistin resistance worldwide and the lack of studies in the Kingdom of Saudi Arabia following this issue [21,22]. These authors encourage investigative studies on colistin resistance in clinical isolates, especially in *A. baumannii* species, to better capture a clear picture of our local epidemiology. Moreover, as we highlighted here, Aruhomukama et al. reported the utility of high-throughput sequencing and bioinformatics in the investigation of colistin resistance [16]. 

In the present study, we conducted genomic characterization of 52 colistin-resistant isolates, including *A. baumannii, K. pneumoniae*, and *P. aeruginosa,* which exhibited a high-resistance phenotype to all tested antibiotics, including some of the last resort drugs such as carbapenems. As well-highlighted in the literature, WGS represents one of the best ways to exhaustively investigate any MDR bacteria’s resistance phenotype and figure out the putative transmission of MDR clones within a given hospital setting. Indeed, here the genome sequencing of all studied isolates allowed us to determine the full resistome of each isolate as well as to exactly identify the colistin resistance mechanism in the latter. In fact, colistin resistance in the three studied bacterial species was mediated by non-synonymous mutations in chromosomal genes associated with colistin resistance [19,23,24], including mainly the *pmrB* gene in *A. baumannii* and *P. aeruginosa* isolates, while in *K. pneumoniae* isolates, these mutations occurred in the PhoP/PhoQ regulator *mgrB, pmrA, pmrB*, and *phoQ* genes. Interestingly, the mobile colistin resistance *mcr*-1 gene was detected on the recombinant plasmid shared by two *K. pneumoniae* isolates. This finding is worrisome and may suggest a beginning of a spread of this transferable resistance in clinical isolates. Moreover, the conducted WGS approach allowed us to deeply investigate the putative dissemination of specific clones within the hospital. While the performed MLST analysis identified isolates belonging to known ST types, such as ST2 in *A. baumannii* isolates, the pairwise comparison of gSNPs between isolates and whole-proteome-based phylogenetic tree analyses allowed us to identify the existence of circulating sub-clones in the three analyzed bacterial species and also several cases of isolate transmissions between hospitalized patients since different isolates collected from different patients shared less than 20 gSNPs, which is suggestive of contamination of patients by the same isolate. These contaminations occurred in the ward settings since patients shared either the same room or the same service. As compared to the literature, the same approach, such as high-throughput sequencing of collection clinical isolates (i.e., *Staphylococcus aureus*) and conducted pairwise comparison of gSNPs, have been reported to described the emergence of the pandemic *S. aureus* clone ST8-USA300 in Geneva, Switzerland [25].

These findings highlight the usefulness of WGS of a collection of isolates to investigate an ongoing outbreak of a bacterial clone or sub-clone. Our result confirms, as reported in the literature, that the *A. baumannii* ST2 clone is the most prevalent ST type of *A. baumannii* found in clinical settings in Saudi Arabia [26]. 

## 4. Materials and Methods

**Sample collection:** Clinical isolates were collected from King Fahd Medical City (KFMC) biobank of KFMC diagnostic microbiology laboratory from December 2012 to 2020 based on their colistin resistance profile as detected by the Phoenix BD system (Becton, Dickinson and Company, USA). Colistin-susceptible isolates were not kept. Moreover, all available metadata (i.e., patient gender, age, year of isolation, and isolation sources) associated with the isolates were also collected. This work and analysis of clinical samples were approved by the Ministry of Health/King Fahad Medical City under the IRB number H-01-R-012. 

**Culture and bacterial identification:** All isolates were cultured on selective MacConkey agar for Gram-negative bacteria (Saudi Prepared Media Laboratory, KSA) and re-identified using matrix-assisted laser desorption and ionization time-of-flight mass spectrometry (MALDI-TOF MS) (Bruker, Germany) [27].

**Antimicrobial susceptibility (AST):** AST profile was recovered from phoenix (since 2012, all bacterial species’ identification and antimicrobial susceptibility have been determined using the automated Phoenix system (BD, US)). The susceptibility test results and minimum inhibitory concentration (MIC) were interpreted according to the CLSI breakpoints criteria (https://clsi.org/standards/products/microbiology/documents/m100, accessed on 14 March 2020). The susceptibility to colistin was evaluated using the E-test (BioMérieux, Marcy l’étoile) and by the broth microdilution method using the ComASPTM (Liofilchem^®^, Roseto degli Abruzzi, Italy) kit according to the manufacturer’s instructions to confirm the CMI obtained for colistin.

**Whole-genome sequencing (WGS):** Bacterial genomic DNA was extracted from all bacterial isolates using DNA extraction mini kit (Pure Link) according to the manufacturer’s instructions (Thermo Fisher Carlsbad, SÁ 92,008 USA). Then, extracted genomic DNA was subjected to genome sequencing using a next-generation sequencing (NGS) approach on the MiSeq platform (Illumina, San Diego, CA, USA) [28]. The Nextera-XT DNA protocol was applied according to the manufacturer’s instructions. Prepared libraries were pooled and diluted to 12 pM, and the balance standard library (PHIX) was spiked at 30% in the diluted pool. Sequencing was performed with MiSeq reagent kit v3 kit (600 cycles). Paired-end reads were demultiplexed into separate samples, and the FastQ files for each sample were retrieved. 

**Genome assembly and WGS analysis:** The quality of the paired-end sequencing reads was assessed using FastQC v.0.11.5 (https://www.bioinformatics.babraham.ac.uk/projects/fastqc/, accessed on 25 May 2020) and trimmed using the Trimmomatic v.0.3835 [29]. Trimmed reads were then subjected to de novo genome assembly using the A5-miseq pipeline [30]. Genome annotation was performed using the prokaryotic genome annotation Prokka, a command line software that fully annotates a bacterial genome in about 10 min [31]. The presence of plasmid sequences in specific genomes was checked using the PlasmidSeeker software [32]. The resistome analysis of all genomes was performed by a command line using ABRicate pipeline v.1.0 (https://github.com/tseemann/abricate, accessed on 25 May 2020), and gene mutations associated with antibiotic resistance were investigated by BlastP and sequence alignment. The genotyping of all isolates was performed by Multilocus sequence typing (MLST) from the genomic sequences, and allelic profiles and sequence types (STs) were assigned according to the corresponding bacterial genus PubMLST scheme. The CVTree3 Web Server was used to investigate the phylogenetic relationship of isolates based on the whole-proteome-based phylogenetic tree [33]. Moreover, to investigate a putative spread of sub-clones within the hospital setting, a pairwise comparison of genomic SNP-based analysis was performed using a local written script with the dnadiff program (part of the Mummer program) [34]. Circular representation of plasmid sequence and its comparison with the closest related plasmids from the NCBI database was generated using the BLAST Ring Image Generator (BRIG) software [35].

## 5. Conclusions

In conclusion, to the best of our knowledge, we report here the first large-scale high-throughput sequencing of clinical isolates combined with genomic analyses in this region, which allowed us to figure out the different mechanisms of antibiotic resistance in different bacterial species. Moreover, while classical molecular analyses such as MLST are limited to exhaustively investigating the diffusion of particular clonal isolates within a given hospital setting, our approach based on WGS and the comparison of the gSNPs of isolates was able to identify circulating sub-clones of isolates and also several cases of isolate transmission between patients. 

## Figures and Tables

**Table 1:** Resistome of the 37 clinical *A. baumannii* isolates in this study.

Isolate Names	ST Type	Resistance to																												
		β-Lactams											Aminoglycosides									Macrolides		Tetracyclines	Sulfonamides	Phenicols	Colistin *			
																											138	142	227	278
																											A	A	A	D
		ADC-73	ADC-30	BlaA1	BlaA2	Mbl	OXA-23	OXA-66	OXA-72	OXA-181	TEM-1D	Zn_hydrolase	ArmA	Aph3’’Ia	AphA6	AadA1-pm	Ant3’’Ih-Aac6-IId	Aac3-I	Aac6-Iaf	StrA	StrB	MphE	MsrE	TetB	SulI	CatB8	PmrB			
AB01	ST604	+	-	+	+	+	+	+	-	-	+	+	+	+	-	-	-	-	-	+	+	+	+	+	-	-	T	.	.	.
AB02	ST2	+	-	+	+	+	+	+	-	-	+	+	+	+	+			-	-	+	+	+	+	+	-	-	T	.	.	G
AB07	ST2	+	-	+	+	+	+	+	-	-	+	+	+	+	+			-	-	+	+	+	+	+	-	-	T	.	.	G
AB08	ST2	+	-	+	+	+	+	+	-	-	+	+	+	+	-	-	-	-	-	+	+	+	+	+	-	-	T	.	.	G
AB12	ST2	+	-	+	+	+	+	+	-	-	+	+	+	+	-	-	-	-	-	+	+	+	+	+	-	-	T	.	.	.
AB14	ST2	+	-	+	+	+	+	+	-	-	-	+	+	+	+	+	+	-	-	+	+	+	+	+	+	+	T	.	V	.
AB15	ST2	+	-	+	+	+	+	+	-	-	+	+	+	+				-	-	+	+	+	+	+	-	-	T	.	.	.
AB16	ST2	+	-	+	+	+	+	+	-	-	+	+	-	+	-	-	-	-	-	+	+	+	+	+	+	-	T	.	.	.
AB17	ST2	+	-	+	+	+	+	+	-	-	-	+	+	+	+	+	+	-	-	+	+	+	+	+	+	+	T	.	V	.
AB18	ST2	+	-	+	+	+	+	+	-	-	-	+	+	-	-	-	-	-	-	+	+	+	+	+	+	-	T	.	.	.
AB19	ST2	+	-	+	+	+	+	+	-	-	+	+	+	+	-	-	-	-	-	+	+	+	+	+	+	-	T	.	.	.
AB20	ST2	+	-	+	+	+	+	+	-	-	-	+	-	+	+	+	+	-	-	+	+	+	+	+	+	+	T	V	.	.
AB21	ST2	+	-	+	+	+	+	+	-	-	+	-	+	+	-	-	-	-	-	+	+	+	+	+	+	-	T	.	.	.
AB22	ST2	+	-	+	+	+	+	+	-	-	-	+	+	-	-	-	-	-	-	+	+	+	+	+	+	-	T	.	.	.
AB23	ST2	+	-	+	+	+	+	+	-	-	+	+	+	+	-	-	-	-	-	+	+	+	+	+	+	-	T	.	.	.
AB24	ST2	+	-	+	+	+	+	+	-	-	-	+	+	+	+	+	+	-	-	+	+	+	+	+	+	+	T	.	V	.
AB26	ST2	+	-	+	+	+	+	+	-	-	-	+	+	-	-	-	-	-	-	+	+	+	+	+	+	-	T	.	.	.
AB29	ST45	-	+	+	+	+	-	+	+	-	-	+	-	-	-	+	-	+	+	-	-	+	+	-	+	-	.	V	.	.
AB30	ST2	+	-	+	+	+	+	+	-	-	-	+	+	-	+	-	-	-	-	+	+	+	+	+	+	-	T	.	.	.
AB33	ST2	+	-	+	+	+	+	+	-	-	-	+	+	-	-	-	-	-	-	+	+	+	+	+	+	-	T	.	.	.
AB54	ST2	+	-	+	+	+	+	+	-	-	+	+	+	+	+	-	-	-	-	+	+	+	+	+	-	-	T	.	.	G
AB191	ST2	-	-	+	+	+	+	+	-	-	-	+	-	-	-	-	-	-	-	+	+	+	+	+	+	-	T	.	.	.
AB192	ST2	+	-	+	+	+	+	+	-	-	-	+	+	-	+	-	-	-	-	+	+	+	+	+	+	-	T	.	.	.
AB193	ST2	+	-	+	+	+	+	+	-	-	-	+	+	-	+	-	-	-	-	+	+	+	+	+	+	-	T	.	.	.
AB194	ST2	+	-	-	+	+	+	+	-	-	-	+	-	-	+	-	-	-	-	+	+	-	-	+	+	-	T	.	.	.
AB195	ST2	+	-	-	+	+	+	+	-	-	-	+	+	-	+	-	-	-	-	+	+	+	+	+	+	-	T	.	.	.
AB196	ST2	+	-	+	+	+	+	+	-	-	+	+	+	+	-	-	-	-	-	+	+	+	+	+	+	-	T	.	.	.
AB197	ST2	+	-	-	+	+	+	+	-	+	-	+	+	-	-	-	-	-	-	+	+	+	+	+	+	-	T	.	.	.
AB198	ST2	+	-	+	+	+	+	+	-	-	-	+	+	-	+	-	-	-	-	+	+	+	+	+	+	-	T	.	.	.
AB199	ST2	+	-	-	+	+	+	+	-	-	-	+	-	-	+	-	-	-	-	+	+	-	-	+	+	-	T	.	.	.
AB201	ST2	+	-	-	+	+	+	+	-	-	-	+	-	-	-	-	-	-	-	+	+	+	-	+	+	-	T	.	.	.
AB202	ST2	+	-	-	+	+	+	+	-	-	-	+	+	-	-	-	-	-	-	+	+	+	+	+	+	-	T	.	.	.
AB203	ST2	+	-	-	+	+	+	+	-	-	-	+	+	-	-	-	-	-	-	+	+	+	+	+	+	-	T	.	.	.
AB204	ST2	+	-	+	+	+	+	+	-	-	-	+	+	-	+	-	-	-	-	+	+	+	+	+	+	-	T	.	.	.
AB1911	ST2	+	-	+	+	+	+	+	-	-	-	+	+	-	-	-	-	-	-	-	-	+	+	-	-	-	T	.	.	.
AB1912	ST2	+	-	-	+	+	+	+	-	-	-	+	+	-	-	-	-	-	-	+	+	+	+	+	+	-	T	.	.	.
AB1913	ST2	+	-	-	+	+	+	+	-	-	-	+	+	-	-	-	-	-	-	+	+	+	+	+	+	-	T	.	.	.

* Associated colistin resistance proteins were compared to those of susceptible A. baumannii ABIsac_ColiS isolates (CAKA00000000.1). No mutation was detected in the other associated-colistin resistance genes, including pmrA, lpxA, lpxC, and lpxD.

**Table 2:** Resistome of the 10 clinical *K. pneumoniae* isolates in this study.

Isolate	ST	β-Lactams										Aminoglycosides						Quinolones				Macrolides			PHE		FOS	SUL	TET			TRP		Colistin *				
		AmpH	TEM-150	OXA-1	OXA-181	SHV-106	SHV-110	CTX-M-15	CTX-M-9	OXA-48	PBP	AadA2	Ant3″Ih	Aac3-IIa	Aph3-VIb	ArmA	StrAB	Sat-2A	OqxA	OqxBgb	QnrB1	MphD	MphE	MsrE	CatA1	CatB4	FosA2	SulI	Tet-34	TetA	TetD	DfrA12	DfrA14	Mcr-1	MgrB	PmrA	PmrB	PhoQ
KP01	873	+	+	+	-	-	+	+	-	+	+	-	+	+	+	-	+	-	+	+	+	+	-	-	-	+	+	+	+	+		-	-	-	Q22P	-	-	-
KP02	873	+	+	+	-	-	+	+	-	+	+	-	+	+	+	-	+	-	+	+	+	+	-	-	-	+	+	+	+	+		-	-	-	Q22P	-	-	-
KP03	2096	+	-	+	+	+	-	-	-	-	+	-	+	-	-	-	-	+	+	+	-	+	-	-	-	+	+	-	+	-	-	-	-	-	-	G53V	-	-
KP04	14	+	-	+	+	+	-	+	-	-	+	+	+	-	-	+	-	+	+	+	-	+	+	+	+	+	+	+	+	-	-	+	-	-	ΔMgrB	-	-	-
KP05	2096	+	+	+	+	+	-	+	-	-	+	+	+	-	-	-	-	+	+	+	-	+	-	+	-	+	+	+	+	-	+	+	-	+	-	-	-	L52V
KP06	2096	+	+	+	-	+	-	+	+	+	+	+	+	-	-	+	-	+	+	+	-	-	+	+	+	+	+	+	+	-	+	+	+	-	-	-	-	-
KP07	2096	+	+	+	+	+	-	+	-	-	+	+	+	-	-	+	-	+	+	+	-	+	+	+	-	+	+	+	+	-	+	+	-	-	-	-	-	G385S
KP08	2096	+	+	+	+	+	-	+	-	-	+	+	+	-	-	+	-	+	+	+	-	+	+	+	-	+	+	+	+	-	+	+	-	-	-	-	T128P; T157P	D495-
KP15	2096	+	+	+	+	+		+	-	-	+	+	+	-	-	-	-	+	+	+	-	+	+	+	-	+	+	+	+	-	+	+	-	+	-	-	-	L52V
KP201	14	+	-	+	+	+		+	-	-	+	+	+	-	-	-	-	+	+	+	-	+	+	+	+	+	+	+	+	-	-	+	-	-	ΔMgrB	-	-	-

PHE: phenicols; FOS: fosfomycin; SUL: sulfonamides; TET: tetracyclines; TRP: trimethoprim. * These proteins were compared with those of the reference *K. pneumoniae* MGH 78,578 isolate (CP000647.1).

**Table 3:** Resistome of the five clinical *P. aeruginosa* isolates in this study.

Resistance to																						
	ST Type	Β-LACT						AGLY					QNE		PHE	SUL	TET	COL *				
		AQU1	OXA-10	OXA-50	GES-1	VEB-1	OprD	AadA1-pm	AadB	Aph3-IIb	Aph6-I	AphA15	4 Copies of OqxBgb	QepA	CatB7	SulI	TetA	Tcr-3	TetR	PmrA	PmrB	PhoQ
PA24	357	+	+	+	-	+	ΔOprD	+	+	+	+	-	+	+	+	+	+	+	+	-	S2P; A4T; V15I; G68S; Y345H	-
PA31	235	+	-	+	+	-	Loss	-	-	+	+	+	+	+	+	+	-	+	-	L71R	S2P; A4T; W25C; G68S; Y345H; G362S	Y85F
PA191	357	+	+	+	-	+	ΔOprD	+	-	+	+	-	+	+	+	+	+	-	+	-	S2P; A4T; V15I; G68S	-
PA193	357	+	+	+	-	+	ΔOprD	+	+	+	+	-	+	+	+	+	+	-	+	-	S2P; A4T; V15I; G68S; L96V; Y345H; G419S	ΔPhoQ
PA201	Unknown	+	-	+	-	-	ΔOprD	-	-	+	+	-	+	+	+	-	-	+	-	L71R	A4T; G131D; Y345H; P369A; D461N; A462T	-

Β-LACT: β-lactams; AGLY: aminoglycosides; QNE: quinolones; PHE: phenicols; SUL: sulfonamides; TET: tetracyclines; COL: colistin. * These proteins were compared with those of the reference *P. aeruginosa* PAO1 isolate.

## Data Availability

All the Whole-Genome Shotgun projects have been deposited at DDBJ/ENA/GenBank under the accession numbers given in Supplementary Table S3.

## Supplementary Materials

The following supporting information can be downloaded at: https://www.mdpi.com/article/10.3390/antibiotics11111597/s1. Supplementary Table S1: The antibiotic susceptibility testing results and the associated clinical metadata of non-fermenter isolates (*A. baumannii* and *P. aeruginosa*). Supplementary Table S2: The antibiotic susceptibility testing results and the associated clinical metadata of *K. pneumoniae* isolates. Supplementary Table S3: General features and GenBank accession numbers of sequenced genomes in this study.
