# Supplementary material for: Genomic Characterization of Colistin-Resistant Isolates from the King Fahad Medical City, Kingdom of Saudi Arabia
Source: Antibiotics (Basel). 2022 Nov 11;11(11):1597. doi: 10.3390/antibiotics11111597 (PMC9686821; doi:10.3390/antibiotics11111597)
Supplement: Supplementary file 1 [file antibiotics-11-01597-s001.zip › antibiotics-1982269-supplementary/Supplementary_files/Suppl. Table S1.pdf]

Suppl. Table S1: The antibiotic susceptibility testing results and the associated clinical metadata of non-fermenter isolates (*A. baumannii* and *P. aeruginosa*)

Strain	Age (Years)	Gender	Year	Isolation sources	MIC of COL (ug/ml)	CAZ	FEP	TZP	MEM	IMP	GN	AMK	SXT	COL	TGC
***A. baumannii* isolates**															
AB1	77	M	2013	Sputum	16	R	R	R	R	R	R	R	R	R	-
AB2	13	F	2013	Endotracheal	16	R	R	R	R	R	R	R	R	R	R
AB7	41	F	2013	Sputum	16	R	R	R	R	R	R	R	R	R	R
AB8	13	F	2013	Wound	8	R	R	R	R	R	R	R	R	R	-
AB12	59	F	2015	Wound	16	R	R	R	R	R	R	R	R	R	R
AB14	76	F	2016	Sputum	16	R	R	R	R	R	R	R	R	R	R
AB15	65	F	2016	Sputum	16	R	R	R	R	R	R	R	R	R	R
AB16	54	M	2016	Sputum	16	R	R	R	R	R	R	R	R	R	R
AB17	18	M	2016	Sputum	16	R	R	R	R	R	R	R	R	R	R
AB18	40	F	2016	Urine	16	R	R	R	R	R	R	R	R	R	R
AB19	40	F	2016	Endotracheal	16	R	R	R	R	R	R	R	R	R	R
AB20	85	M	2016	Blood	16	R	R	R	R	R	R	R	R	R	R
AB21	50	F	2016	Wound	16	R	R	R	R	R	R	R	R	R	R
AB22	48	M	2016	Sputum	16	R	R	R	R	R	R	R	R	R	R
AB23	62	F	2016	Sputum	16	R	R	R	R	R	R	R	R	R	R
AB24	50	F	2016	Blood	16	R	R	R	R	R	R	R	R	R	R
AB26	99	M	2017	Sputum	16	R	R	R	R	R	S	S	R	R	R
AB29	89	F	2018	Sputum	16	R	R	R	R	R	R	R	R	R	R
AB30	58	M	2018	Wound	16	R	R	R	R	R	R	R	R	R	R
AB33	86	M	2018	Sputum	16	R	R	R	R	R	S	S	R	R	R
AB54	55	M	2019	Sputum	16	R	R	R	R	R	R	R	R	R	R
AB191	26	F	2019	Rectal swab	8	R	R	R	R	R	S	S	R	R	-
AB192	22	M	2019	Peripheral line	64	R	R	R	R	R	R	R	R	R	-

AB193	68	M	2019	Indwelling catheter	128	R	R	R	R	R	R	R	R	R	R
AB194	27	M	2019	Endotracheal	32	R	R	R	R	R	R	R	R	R	R
AB195	21	F	2019	Cerebrospinal fluid	16	R	R	R	R	R	R	R	R	R	-
AB196	21	F	2019	Cerebrospinal fluid	32	R	R	R	R	R	R	R	R	R	R
AB197	74	F	2019	Bedsores	16	R	R	R	R	R	S	S	R	R	R
AB198	76	M	2019	Sputum	32	R	R	R	R	R	R	R	R	R	R
AB199	75	M	2019	Sputum	16	R	R	R	R	R	R	R	R	R	R
AB201	77	F	2020	Sputum	16	R	R	R	R	R	R	R	R	R	R
AB202	57	M	2020	Sputum	16	R	R	R	R	R	S	S	R	R	R
AB203	81	M	2020	Urine	16	R	R	R	R	R	S	S	R	R	R
AB204	60	F	2020	Endotracheal	32	R	R	R	R	R	R	R	R	R	R
AB1911	18	M	2019	Bedsores	128	R	R	R	R	R	S	S	R	R	R
AB1912	80	M	2019	Sputum	32	R	R	R	R	R	S	S	R	R	R
AB1913	57	M	2019	Central venous	32	R	R	R	R	R	S	S	R	R	R
***P. aeruginosa* isolates**															
PA24	31	M	2017	Wound	16	R	R	R	R	R	R	R	R	R	-
PA31	28	M	2018	Sputum	4	R	R	R	R	R	R	S	R	R	R
PA191	32	M	2019	Sputum	128	R	R	R	R	R	R	R	R	R	-
PA193	32	M	2019	Sputum	128	R	R	R	R	R	R	R	R	R	R
PA201	77	F	2020	Sputum	4	R	R	R	R	R	R	R	R	R	-

M: Male; F: female; CAZ: ceftazidime; FEP: cefepime; TZP: ticarcillin/tazobactam, MEM: meropenem; IMP: imipenem; GN: gentamicin; AMK: amikacin; SXT: trimethoprim/sulfamethoxazole; COL: colistin; TGC: tigecycline; R: Resistant; S: susceptible; ND: not determined.
