# Supplementary material for: Genomic Characterization of Colistin-Resistant Isolates from the King Fahad Medical City, Kingdom of Saudi Arabia
Source: Antibiotics (Basel). 2022 Nov 11;11(11):1597. doi: 10.3390/antibiotics11111597 (PMC9686821; doi:10.3390/antibiotics11111597)
Supplement: Supplementary file 1 [file antibiotics-11-01597-s001.zip › antibiotics-1982269-supplementary/Supplementary_files/Suppl. Table S2.pdf]

Suppl. Table S2: The antibiotic susceptibility testing results and the associated clinical metadata of *K. pneumoniae* isolates

Strains	Age (Years)	Gender	Years	Site	MIC of COL (ug/ml)	AMC	CF	TZP	CRO	CFX	CAZ	FEP	ETP	MEM	IMP	GN	AMK	COL	SXT	CIP	TGC	LVX
**KP1**	72	F	2017	Blood	16	R	R	R	R	R	R	R	R	R	R	R	R	R	R	R	R	R
**KP2**	83	F	2017	Urine	8	R	R	R	R	R	R	S	R	R	R	R	R	R	R	R	R	R
**KP3**	76	F	2018	Sputum	16	R	R	R	R	R	R	R	R	R	R	R	R	R	R	R	R	R
**KP5**	41	F	2018	Sputum	8	R	R	R	R	R	R	R	R	R	R	R	R	R	R	R	R	R
**KP15**	41	F	2019	Blood	16	R	R	R	R	R	R	R	R	R	R	R	R	R	R	R	R	R
**KP6**	51	M	2019	Sputum	32	R	R	R	R	R	R	R	R	R	R	R	R	R	R	R	R	R
**KP7**	89	M	2019	Tissue	128	R	R	R	R	R	R	R	R	R	R	R	R	R	R	R	R	R
**KP8**	89	F	2019	Wound	16	R	R	R	R	R	R	R	R	R	R	R	R	R	R	R	R	R
**KP4**	73	M	2018	Endotracheal	16	R	R	R	R	R	R	R	R	R	R	R	R	R	R	R	R	R
**KP201**	27	M	2020	Sputum	128	R	R	R	R	R	R	R	R	R	R	S	R	R	R	R	I	R

M: Male; F: female; AMC: amoxicillin/clavulanic acid; CF: cefalotin; TZP: ticarcillin/tazobactam; CRO: ceftriaxone; CFX: cefuroxime; CAZ: ceftazidime; FEP: cefepime; ETP: ertapenem; MEM: meropenem; IMP: imipenem; GN: gentamicin; AMK: amikacin; COL: colistin; SXT: trimethoprim/sulfamethoxazole; CIP: ciprofloxacin; TGC: tigecycline; LVX: levofloxacin; R: Resistant; S: susceptible; I: Intermediate.
