# Supplementary material for: Genomic Characterization of Colistin-Resistant Isolates from the King Fahad Medical City, Kingdom of Saudi Arabia
Source: Antibiotics (Basel). 2022 Nov 11;11(11):1597. doi: 10.3390/antibiotics11111597 (PMC9686821; doi:10.3390/antibiotics11111597)
Supplement: Supplementary file 1 [file antibiotics-11-01597-s001.zip › antibiotics-1982269-supplementary/Supplementary_files/Suppl. Table S3.pdf]

Suppl. Table S3 : General features and Genbank accession numbers of sequenced genomes in this study

Organisms	Isolate name	Number of contigs	Genome Size (bp)	% GC content	Coverage (X)	Genbank Accession number
A. baumannii	AB1	173	3968461	39	64,9	JALKQN000000000
A. baumannii	AB02	283	3994903	38,9	107,43	JALKQO000000000
A. baumannii	AB07	102	4006090	38,8	165,36	JALKQP000000000
A. baumannii	AB8	143	3920431	39	65,5	JALKQQ000000000
A. baumannii	AB12	269	3946766	39	80,21	JALKQR000000000
A. baumannii	AB14	191	3962960	38,9	77,88	JALKQS000000000
A. baumannii	AB15	99	4009228	38,8	163,33	JALKQT000000000
A. baumannii	AB16	267	3961410	38,9	97,71	JALGJE000000000
A. baumannii	AB17	110	3963639	38,9	105,15	JALGJF000000000
A. baumannii	AB18	123	3908466	38,9	39,47	JALGJG000000000
A. baumannii	AB19	111	3984215	38,8	29,41	JALGJH000000000
A. baumannii	AB20	551	3840579	39,2	73,69	JALGJI000000000
A. baumannii	AB21	353	3936606	39	19,19	JALGJJ000000000
A. baumannii	AB22	376	3838504	39,1	15,19	JALGJK000000000
A. baumannii	AB23	113	3988477	38,8	48,87	JALGJL000000000
A. baumannii	AB24	149	3959677	38,9	24,44	JALGJM000000000
A. baumannii	AB26	134	3916613	38,9	82,06	JALGJN000000000
A. baumannii	AB29	369	3776738	39,1	35,09	JALGJO000000000
A. baumannii	AB30	144	3993169	38,8	40,59	JALGJP000000000
A. baumannii	AB33	300	3865574	39	94,46	JALGJQ000000000
A. baumannii	AB54	192	4016088	38,9	175,84	JALGJR000000000
A. baumannii	AB191	503	3813207	39,2	97,41	JALGJS000000000
A. baumannii	AB192	194	3954659	38,8	128,38	JANZML000000000
A. baumannii	AB193	217	3981643	38,9	62,37	JANZMM000000000
A. baumannii	AB194	134	3933232	38,8	98,75	JANZMN000000000
A. baumannii	AB195	164	3998162	38,8	100,03	JANZMO000000000

A. baumannii	AB196	142	4022628	38,8	98,62	JANZMP000000000
A. baumannii	AB197	153	3877426	38,8	79,03	JANZMQ000000000
A. baumannii	AB198	141	4003719	38,8	87,06	JANZMR000000000
A. baumannii	AB199	121	3932205	38,8	77,4	JANZMS000000000
A. baumannii	AB201	812	3788285	39,3	25,19	JANZMT000000000
A. baumannii	AB202	200	3969627	38,9	119,46	JANZMU000000000
A. baumannii	AB203	258	3873879	39	168,27	JANZMV000000000
A. baumannii	AB204	140	3919537	38,9	145,05	JANZMW000000000
A. baumannii	AB1911	128	3876481	38,9	119,65	JANZMX000000000
A. baumannii	AB1912	306	3988172	38,8	86,05	JANZMY000000000
A. baumannii	AB1913	197	3860717	38,9	121,48	JANZMZ000000000
P. aeruginosa	PA24	81	6686118	65,9	85,07	JANZNK000000000
P. aeruginosa	PA31	79	6865610	66	63,66	JANZNL000000000
P. aeruginosa	PA191	1484	7699524	65,8	23,77	JANZNM000000000
P. aeruginosa	PA193	554	6531916	65,9	14,49	JANZNN000000000
P. aeruginosa	PA201	134	6644905	66,2	89,98	JANZNO000000000
K. pneumoniae	KP01	128	5702762	57	80,25	JANZNA000000000
K. pneumoniae	KP02	72	5714812	57	107,55	JANZNB000000000
K. pneumoniae	KP03	75	5425084	57,3	71,13	JANZNC000000000
K. pneumoniae	KP04	105	5809953	56,7	47,73	JANZND000000000
K. pneumoniae	KP05	207	5708925	56,7	80,87	JANZNE000000000
K. pneumoniae	KP06	117	5949533	56,5	24,21	JANZNF000000000
K. pneumoniae	KP07	98	5756680	56,7	25,28	JANZNG000000000
K. pneumoniae	KP08	165	5733557	56,7	20,17	JANZNH000000000
K. pneumoniae	KP15	108	5775816	56,6	111,19	JANZNI000000000
K. pneumoniae	KP201	151	5846061	56,7	61,73	JANZNJ000000000
